# A comprehensive proteomics profiling identifies NRP1 as a novel identity marker of human bone marrow mesenchymal stromal cell-derived small extracellular vesicles

**DOI:** 10.1186/s13287-019-1516-2

**Published:** 2019-12-18

**Authors:** Afnan Munshi, Jelica Mehic, Marybeth Creskey, Jonathan Gobin, Jun Gao, Emma Rigg, Gauri Muradia, Christian C. Luebbert, Carole Westwood, Andrew Stalker, David S. Allan, Michael J. W. Johnston, Terry Cyr, Michael Rosu-Myles, Jessie R. Lavoie

**Affiliations:** 1 Centre for Biologics Evaluation, Biologics and Genetic Therapies Directorate, Health Products and Food Branch, Health Canada, Ottawa, Ontario Canada; 2 University of Ottawa, Ottawa, Ontario Canada; 3 University of Carleton, Ottawa, Ontario Canada

**Keywords:** Human bone marrow-derived mesenchymal stromal cells, Paracrine effectors, Small extracellular vesicles, Proteomics, Pathway enrichment, Identity marker

## Abstract

**Background:**

Clinical applications have shown extracellular vesicles (EVs) to be a major paracrine effector in therapeutic responses produced by human mesenchymal stromal/stem cells (hMSCs). As the regenerative capacity of EVs is mainly ascribed to the transfer of proteins and RNA composing its cargo, and to the activity attributed by the protein surface markers, we sought to profile the protein composition of small EVs released from hMSCs to identify hMSC-EV biomarkers with potential clinical relevance.

**Methods:**

Small EVs were produced and qualified from five human bone marrow MSC donors at low passage following a 48-h culture in exosome-depleted medium further processed by steps of centrifugation, filtration, and precipitation. Quantitative proteomic analysis comparing the protein profile of the EVs released from hMSCs and their parental cell was conducted using tandem mass tag labeling combined to mass spectrometry (LC-MS/MS) to identify enriched EV protein markers.

**Results:**

Nanoparticle tracking analysis showed no differences in the EV concentration and size among the five hMSC donors (1.83 × 10^10^ ± 3.23 × 10^9^/mL), with the mode particle size measuring at 109.3 ± 5.7 nm. Transmission electron microscopy confirmed the presence of nanovesicles with bilayer membranes. Flow cytometric analysis identified commonly found exosomal (CD63/CD81) and hMSC (CD105/CD44/CD146) markers from released EVs in addition to surface mediators of migration (CD29 and MCSP). Quantitative proteomic identified 270 proteins significantly enriched by at least twofold in EVs released from hMSCs as compared to parental hMSCs, where neuropilin 1 (NRP1) was identified among 21 membrane-bound proteins regulating the migration and invasion of cells, as well as chemotaxis and vasculogenesis. Validation by western blot of multiple batches of EVs confirmed consistent enrichment of NRP1 in the nanovesicles released from all five hMSC donors.

**Conclusion:**

The identification and verification of NRP1 as a novel enriched surface marker from multiple batches of EVs derived from multiple hMSC donors may serve as a biomarker for the assessment and measurement of EVs for therapeutic uses.

## Introduction

From a clinical perspective, the ability of human mesenchymal stromal/stem cells (hMSCs) to produce potent inhibitory effectors influencing both the innate and adaptive immune system as well as their capacity to release anti- and pro-inflammatory effectors has rendered them a popular source of cells for treating acute tissue injury syndromes, chronic degenerative disorders, and inflammatory diseases. The most prevalent source of hMSCs in clinical trials is adult bone marrow (BM) where they present a low immunogenicity profile granted by their constitutively low expression levels of MHC class I and HLA I and lack of expression of MHC class II and HLA-DR molecules [1]. These characteristics along with their reported clinically safe profile have propelled their use for allogeneic/autologous transplantation in a wide range of diseases [2].

Whole-cell therapy, including hMSC therapy, is an important therapeutic option for treating diseases in a variety of clinical contexts that require transplanted cells to survive in the treated area by integrating into the native tissue to replace the injured cells and/or to stimulate the endogenous tissue repair mechanism. For example, in the treatment of traumatic articular cartilage defects, hMSC differentiation-based tissue engineering techniques have been proposed to resurface articular cartilage defects and induce cartilage regeneration [3]. However, when the mechanism of action that leads to the clinical outcome depends primarily on the secretome of the stem cell (i.e., released growth factors, chemokines, cytokines, morphogens, small molecules, and extracellular vesicles), rather than the cellular differentiation and replacement mechanisms, cell-free therapeutic approaches may be prioritized [4]. Towards this, cell-free therapy based on extracellular vesicles is being investigated as a therapeutic strategy for treating diseases where adjacent and distant responder cells as well as tissue activity are affected by the paracrine effectors [5–7]. Extracellular vesicles (EVs) are lipid bilayer vesicles ranging in size of roughly from 40 to 1000 nm in diameter, which have important roles in communication and transportation between cells [8]. In particular, small EVs (sEVs; 50–200 nm diameter) represent a population of cargo-bearing vesicles (proteins, microRNA/mRNA, lipids) important for cell biology processes and are now regarded as new therapeutic agents that are proposed for testing in clinical trials [9]. The terms sEVs and exosomes are often interchangeably used for describing sEVs, but since the exosomes’ nomenclature requires specific evidence of endosomal biogenesis pathway, the term sEV has been recommended [4].

The cargo-bearing sEVs released by hMSCs, for example, contain MSC-associated critical surface markers and signaling molecules characteristic of the parental cell, thereby potentially mediating the therapeutic effects of the MSCs [10]. This notion of MSC paracrine-mediated therapeutic effect has been reported in many studies where low cell engraftment after systemic administration of MSCs was described, while clinical effectiveness was still achieved [11–13]. Numerous pre-clinical studies demonstrating the regenerative capacities of MSCs by secreted factors alone in a paracrine rather than a cellular manner in the treatment of acute tissue injury syndromes for kidney [14], myocardial [15, 16], cerebral [17–20], and liver tissues [21–24] have been published. Furthermore, human therapeutic testing of hMSC-derived small EVs has started. Three clinical trials have been registered to date on the Clinicaltrials.gov international database for self-reported industry-sponsored trials for treating acute ischemic stroke (NCT03384433), chronic kidney disease and type I diabetes mellitus (NCT02138331), and macular holes (NCT03437759), in addition to one published report describing the treatment regimen of a patient suffering from graft-versus-host disease (GvHD) [25]. The clinical use of sEVs as opposed to the cell itself as the therapeutic addresses many of the challenges observed with whole-cell therapy. Due to their small size (< 200 nm in diameter) sEVs do not occlude the microvasculature upon intravenous injection and can be filter sterilized [26]. Furthermore, in contrast to cells, a cell-derived EV lacks a nucleus, which means that it cannot self-replicate and therefore present lower tumorigenic potential. This characteristic also means that the injected sEVs lack the ability to respond to a microenvironment once transplanted as opposed to cells where unexpected reactivity can be observed upon injection as cells have the ability to respond to and to be shaped by local inflammatory conditions for example [1]. However, a safety aspect to consider for EV therapeutic is related to the cargo-bearing constituents of an EV which reflect the producer cells, which means that EVs have the potential to carry a tumorigenic-promoting cargo if produced by a tumor or transformed cell [27, 28]. To enable the safe and efficacious development of MSC-sEVs as therapeutics, it is therefore critical to decipher its molecular composition in order to define and qualify the EVs for therapeutic purposes. The importance of interpreting the molecular composition of EVs also lies in their ability to modulate recipient cell signaling and mediate cell-to-cell communication via receptor-mediated interaction and delivery of their cargo-bearing components. Following the EV-recipient cell interaction, EVs can be internalized by endocytosis where they can transfer their payload of proteins, mRNA, miRNA, lipids, and metabolites via fusion of EV-cell membranes [29]. Importantly, EVs can stimulate cell signaling pathways of the recipient cells by receptor-mediated interaction without the transfer of cargo-bearing bioactive molecules [30]. As well, EVs can transfer surface receptors or lipids to the recipient cells, thereby modulating the host cell [29]. Ensuring consistency and quality of the EV therapeutics is of tremendous importance, as its molecular composition will reflect the environment in which it was produced/manufactured, and can therefore dictate the clinical outcome. For example, a high abundance of pro-inflammatory cytokines in EVs may stimulate cancer cell metastasis rather than controlling its growth [31, 32].

Several sEV isolation techniques have been utilized, differential centrifugation being one of the most commonly used method, but due to its low scalability and potential loss of EV membrane integrity is less attractive for large-scale studies [33]. The density gradient centrifugation method of EV isolation yields the highest purity but at the expense of scalability, yield, cost, time, and therapeutic potency [33]. Other popular methods include, immunoaffinity methods using exosomal tetraspanin markers [34], size exclusion chromatography (SEC), and EV purification in closed systems such as tangential flow filtration (TFF) [33, 35]. Polyethylene glycol (PEG)-based precipitation of EVs is also a popular method of isolation as it is highly scalable and easy to use; however, some studies have reported that the EV preparation can be contaminated with lipoparticles and other vesicles of similar size [36]. To this date, the only published MSC-EV therapeutic human trial used the PEG-based method and showed promising clinical outcome for a patient with steroid refractory GvHD [25].

Albeit many reports have described the testing of EVs, there are currently no gold standard methods for sEV purification, characterization, and quantification or in vitro potency assay available. However, the International Society of Extracellular Vesicle (ISEV) has published in 2014 and 2018 minimal guidelines for EV reporting to increase reproducibility in the field [37, 38]. Therefore, in order to provide a substantial contribution to the EV therapeutics field, the EV studies should accurately identify/characterize the producer cell of the EVs and the presence of lipid-membrane vesicles, as well as describe the physical and molecular properties of the vesicles. Furthermore, no quality control assays are currently in place to determine safety and efficacy of sEV therapeutics, highlighting the importance of performing comprehensive molecular profiling of sEVs, including protein characterization, to address this. Indeed, despite the increasing evidence of MSC-EV proteins as the main driver of the therapeutic effects in many studies, only a few reports addressed this. For example, Yuan and colleagues conducted a thorough investigation of the enriched protein profile by LC-MS/MS of hMSC-EVs obtained from primed MSCs grown under low oxygen conditions [39]. These proteomics studies, including the one described here, will provide potential candidate MSC-EV proteins that will lead to quantifiable features, as well as reproducible and standardized assays.

In order to fulfill this objective, identification of proteins composing qualified MSC-sEVs and their participation into pathway activation/inhibition will help identify MSC-sEVs and may ultimately elucidate the sEV’s mechanism of action. We report a comprehensive phenotypic characterization as well as comparative quantitative mass spectrometry-based proteomic analysis of human MSC-sEVs derived from five different hBM-MSC donors where the parental cell protein profile was compared to their corresponding sEVs to identify enriched proteins. The identification and verification of NRP1 as an enriched surface marker protein on MSC-sEVs from multiple batches of donor samples may serve as an EV biomarkers for the assessment and measurement of MSC-EVs for therapeutics uses.

## Material and methods

### hBM-MSC culture expansion

Human bone marrow mesenchymal stromal/stem cells (hBM-MSCs) derived from five healthy male hBM donors (ages 22–28 years old) were characterized according to the ISCT’s minimal criteria [40] and obtained from the Texas A&M Health Science Center College of Medicine Institute for Regenerative Medicine at Scott & White through a grant from ORIP of the NIH, Grant # P40OD011050 (Additional file 4: Table S1). The bone marrow donors recruited by the Texas A&M Health Science Center College of Medicine were normal healthy adults at least 18 years of age, not presenting any of these exclusion criteria (pregnant; chronic illness such as diabetes; on prescription medication; body mass index lower or higher than average; history of cancer, tumors or abnormal growths; genetic diseases; bleeding disorders; and allergies to lidocaine). hBM-MSCs were expanded to generate working cell banks as per Texas A&M University Health Science Center’s protocol [37] with slight modifications described herein. In brief, hBM-MSCs were seeded at 1 × 10^6^ cells in T-175 flasks overnight as per recommendation, then harvested using 0.25% Trypsin-EDTA (Gibco, Cat#25200-072) in the morning and re-seeded at a seeding density of 17,500 cells/T-175 (100 cell/cm^2^), as per recommendation. hBM-MSCs were cultured in alpha-MEM (Invitrogen, Cat#12561-056) supplemented with 15% MSC-screened FBS (HyClone, Cat#SH30070.03) in T-175 flasks for cell expansion. Culture medium was changed on day 4, and cells were harvested and frozen on day 7 to create working cell banks for our experiments.

### hBM-MSC-sEV production

A 7-day culture timeline for hBM-MSC expansion was established and this included a 48-h EV production time. hBM-MSCs were initially seeded at an optimized seeding density of 1.4 × 10^5^ per T-175 flask on day 1 in 25 mL of alpha-MEM supplemented with 15% MSC-screened FBS (HyClone, Cat#SH30070.03) (i.e., complete medium). On day 4, the culture medium was replaced with fresh 25 ml of complete medium for ensuring good cell health. On day 5, a culture medium switch was performed to remove exosomal bovine contaminants from the FBS prior to commencing hBM-MSC-EV production: medium was aspirated, cells were rinsed twice with 15 mL of PBS, and 32 mL of alpha-MEM supplemented with 15% exosome-depleted FBS (ED-FBS) (Gibco, Cat#A2720801) was added per flask. At day 7, the cell-conditioned media (CCM) was collected separately for each flask, centrifuged at 2000×*g* for 30 min to remove cell debris, and the CCM supernatant was frozen at − 80 °C. Thereafter, cells were harvested and live cell counts were recorded to later normalize EV counts per live cell. After the counts, cells were rinsed twice with cold PBS by centrifuging at 300×*g* for 8 min, and after the second rinse, the PBS was aspirated and the cell pellet was stored at − 80 °C for future use.

### hBM-MSC-sEV isolation

The 15-mL CCM of each hBM-MSC sample was thawed at room temperature on the day of use and processed immediately once liquid while still cold (Additional file 1: Figure S1). Each CCM aliquot was filtered using a 0.2-μm PALL Acrodisc 25 mm syringe filter (Pall, Cat#4612) and was then added to an Amicon Ultra-15 Centrifugal filters Ultra cel-10 K (Millipore, Cat# UFC901024) (previously washed and equilibrated with PBS according to the company’s protocol) and centrifuged at 2000×*g* for 20 min. The Amicon collection tube was emptied of filtrate, and filtered PBS (PBS filtered using a 0.2 μm PALL Acrodisc 25 mm syringe filter (Pall, Cat# 4612)) was added to the concentrated CCM sample to obtain a final volume of 15 mL. The sample was then centrifuged a second time at 2000×*g* for 20 min. The concentrated CCM sample was transferred to a new 50 mL Falcon tube mixed with 0.5 volume of Total Exosome Isolation Reagent (Invitrogen, Cat#4478359) and vortexed. The sample was allowed to incubate overnight at 4 °C, and in the morning was centrifuged at 10,000×*g* for 1 h at 4 °C. The supernatant was then removed, and the EV pellet was suspended in filtered PBS.

### Flow cytometry

#### hBM-MSC surface marker analysis

To analyze the expression of hBM-MSC surface markers set by the ISCT’s minimal criteria for MSC characterization, the human MSC analysis kit from BD Biosciences (Cat#562245) was used according to the manufacturer’s protocol (Additional file 2: Figure S2). This kit contains antibodies for MSC positive (CD73, CD90, and CD105) and negative (CD11b, CD19, CD34, CD45, and HLA-DR) surface markers (refer to the manufacturer’s protocol for the antibodies’ specifications). Briefly, hBM-MSCs cultured under the expansion protocol described in the “hBM-MSC culture expansion” section were harvested, washed using PBS + 2% FBS (i.e., flow buffer), counted and suspended in 1 mL of flow buffer followed by a filtration step through a 40-μm cell strainer to remove possible cell clumps. One hundred microliters of cell suspension was then added to each flow tube (0.5 × 10^6^ cells per tube, 9 tubes total as per protocol to which specific antibodies provided in the kit were added). Each tube was incubated in the dark for 30 min at 4 °C after which the cells were washed two times with the flow buffer where the volume was brought up to 4 ml with the flow buffer and cells were centrifuged at 1100 rpm for 6 min at 4 °C. The supernatant was discarded and the pellet was suspended in 500 μL of flow buffer and analyzed by flow cytometry using the LSRII flow cytometer (BD Biosciences). One hundred thousand events per sample were collected, and raw data was analyzed using FlowJo V10 (FlowJo LLC, Ashland, OR, USA).

#### CD63-positive hBM-MSC-sEV surface marker analysis

hBM-MSC-sEVs were analyzed using flow cytometry to confirm the presence of EV/exosome-specific tetraspanin markers CD63, CD81, and CD9. The analysis was conducted using CD63-conjugated 4 μm magnetic beads according to the manufacturer’s protocol (Invitrogen, Cat#10622D), except for the total volume of hBM-MSC-sEVs which was further optimized. hBM-MSC-sEVs were isolated according to the protocol mentioned in the “hBM-MSC-sEV isolation” section. For this procedure, hBM-MSC-sEVs were isolated from a starting volume of 15 mL of hBM-MSC CCM and the hBM-MSC-sEVs were suspended in filtered PBS. The hBM-MSC-sEV suspension is referred to as “pre-enriched hBM-MSC-sEVs.” Briefly, for flow cytometry, isolation buffer (filtered PBS + 0.1% BSA) was prepared and filtered through a 0.2-μm syringe filter. Forty microliters of the magnetic beads was first rinsed with 200 μL of the isolation buffer using the DynaMag2 magnet (Invitrogen, Cat#12321D), and then the washed magnetic beads were incubated with 300 μL of “pre-enriched hBM-MSC-sEVs.” Each sample was then vortexed for 30 s in round bottom 2 mL tubes and incubated overnight at 4 °C while mixing using Orbitron rotator. After incubation, each sample was centrifuged for 30 s at 1000 rpm to gather the bead-bound sEV samples at the bottom of the tube. Bead-bound hBM-MSC-sEVs were then rinsed twice with 300 μL followed by 400 μL of the isolation buffer using the DynaMag2 magnet and removing the supernatant. Bead-hBM-MSC-sEV conjugates were suspended in 500 μL of the isolation buffer from which 100 μL was transferred to each 5 mL flow tubes and stained separately with CD63, CD81, and CD9 antibodies or corresponding isotype IgG1κ. The concentration of the CD63, CD81, and CD9 antibodies was matched to the corresponding isotype antibody concentration. Each sample was incubated for 45 min in the dark while shaking. The stained sample was then rinsed two times with the isolation buffer and the supernatant containing excess antibody was discarded using DynaMag2 magnet. Finally, the sample was suspended in 0.5 mL of the isolation buffer and placed on ice until ready for the flow cytometric analysis using the LSRII flow cytometer (BD Biosciences). Ten thousand events per sample were collected. Raw data was analyzed using FlowJo V10 (FlowJo LLC, USA) where CD63, CD81, and CD9 positive expression was measured against isotype control IgG1κ which served as a negative control. For information on the antibodies, refer to the manufacturer’s protocol (Invitrogen, Cat#10622D).

#### Multiplex bead-based flow cytometric assay of hBM-MSC-EV 37 EV markers

For multiplex bead based, flow cytometric analysis was conducted using 30 mL of hBM-MSC-sEV CCM isolated as described in the “hBM-MSC-sEV isolation” section using the MACSplex Exosome kit (human) (Miltenyi Biotec, Cat#130-108-813), where the EV pellet was suspended in 460 μL of filtered PBS. Following isolation, EV samples were transferred to 1.5 mL Protein LoBind tubes (Eppendorf, Cat#0030.108.116) where 40 μL of MACSplex Exosome Capture Beads were added to each EV sample and incubated overnight. Samples were processed as per manufacturer’s recommendations using the “Overnight protocol for the assay using 1.5 mL tubes”; detection of EVs was done using the CD63 MACSplex Exosome Detection Reagent. Following labelling, samples were transferred to 5 mL FACS tubes (BD Biosciences, Cat# 382058) and analyzed by flow cytometry using the LSRII flow cytometer (BD Biosciences). Ten thousand events per sample were collected. Raw data was analyzed using FlowJo V10 (FlowJo LLC, USA).

### Nanoparticle tracking analysis (NTA) of hBM-MSC-sEVs using NanoSight NS300

For NTA analysis using the NanoSight NS300 (Malvern Panalytical), hBM-MSC-sEVs were isolated from 15 mL of CCM, as described in the “hBM-MSC-sEV isolation” section, where the EV pellet was suspended in 0.5 mL of filtered PBS. Sixty microliters was then used from the 0.5 mL of the hBM-MSC-sEV sample and diluted 50× in filtered PBS to obtain a final volume of 3 mL for analysis. Each sample was vortexed prior to filling the syringe with 1 mL of the 50× diluted hBM-MSC-sEV sample, and the syringe pump from Harvard Apparatus (Cat# 98-4730) was used to run in flow mode. Each 1 mL sample was ran using the following script: six captures of 1 min at speed 10 under flow mode. For capture settings, a camera level of 15 was used for all samples. For analysis settings, a detection threshold of 13 was used for all samples. In between each sample, 3 mL of filtered water (filtered using a 0.2 μm syringe filter), 3 mL of diluted ethanol, and 3 mL filtered water were flushed through the system for cleaning purposes. Analysis of the raw data was performed using Excel and GraphPad Prism 7, where analysis of 5 captures out of 6 was performed, removing the first capture. To generate the approximate total hBM-MSC-sEV concentration per T-175 flask, the dilution factor 50 mentioned above was accounted for, as well as the total volume of 30 ml of CCM as each flask was maintained in 30 mL of culture medium.

### hBM-MSC and hBM-MSC-sEV protein lysate preparation for Western blot and mass spectrometry analysis

Lysis buffer (100 mM TEAB with 1% SDS) was prepared according to the manufacturer’s protocol using the reagents provided in the TMT labeling kit (TMT 10plex Mass Tag Labeling Kits and Reagents Thermo, Cat#90113) (Additional file 7). Two hundred fifty microliters of lysis buffer was added to the cell or EV pellets (EVs isolated from 30 ml of CCM as per the “hBM-MSC-sEV isolation” section) and gently vortexed. RIPA lysis buffer (5×) (Alfa Aesar, Cat# J62524) was also used when preparing sEV samples for Western blot validation for preparing additional EV lysate batches other than the one used for mass spectrometry experiments. The sEV protein samples were incubated for proper lysing for 30 min at 4 °C on over end shaker (LabQuake Shaker) and then centrifuged down for 30 s at 1000 rpm after incubation. The protein supernatants were sonicated (Fisher Scientific, Model#FB120) at amplitude setting 20% (sEVs) or 30% (cells) for 3 × 10 s with 30 s on ice between pulses. After sonication, samples were centrifuged at 14,000×*g* for 5 min at 4 °C. The supernatant was recovered and stored in 1.5 mL Protein LoBind tubes at − 80 °C. Prior to freezing at − 80 °C, an aliquot was taken out for protein quantification by the bicinchoninic acid (BCA) assay (Pierce BCA Protein assay kit, Cat#23227). Samples were kept frozen at − 80 °C for downstream analysis.

### Western blots

When hBM-MSC and hBM-MSC-sEV samples were compared, 40 μg of protein lysates were used. When only samples from the hBM-MSC-EV group were analyzed, 20 μg of protein lysates were used. All samples were combined with 4X LI-COR Protein Loading Buffer (LI-COR, Cat#928-40004) and Bolt™ 10X Sample Reducing Agent (Invitrogen, Cat#B0009) to a final concentration of 1×. Samples were boiled for 5 min then loaded onto precast Bolt™ Bis-Tris 4–12% SDS-PAGE (Invitrogen, Cat#NW04127BOX). Gels were ran using a MOPS buffer system (Invitrogen, Cat#B0001) for 30 min at 200 V. Gels were trimmed and transferred to a Millipore Immobilon FL PVDF membrane (Millipore, Cat#IPFL00005) using the Bolt™ Mini Module wet transfer system for one hour at 20 V. Following transfer, membranes were washed three times in distilled water for 5 min on an orbital shaker at speed 4 (~ 300 rpm) and probed for respective antibody: NRP1 (Anti-Neuropilin 1 antibody EPR3113 RabMab, Abcam, Cat#ab81321), HSP90B1 or GRP94 (Anti-GRP94 antibody EPR3988 RabMab, Cat#ab108606), MMP2 (Anti-MMP2 antibody EPR1184 RabMab, Abcam, Cat#ab92536), GAPDH (Anti-GAPDH antibody, GeneTex Ms mAB GT239, Cat#GTX627408), and secondary antibody IRDye 800CW Goat anti-Rabbit IgG (LI-COR, Cat#925-32211), and IRDye 800CW Goat anti-Mouse IgG Cat#925-32210). Each blot was thereafter stained with LI-COR REVERT total protein stain (LI-COR, Cat#926-11010) according to manufacturer’s protocol and then imaged at 700 nm using the LI-COR Odyssey CLx NIR imager. When GAPDH detection was performed, the blots were stripped using LI-COR NewBlot PVDF Stripping buffer 5X (LI-COR, Cat#928-40032) according to the manufacturer’s procedure. Membranes were blotted using the iBind Western Device (Invitrogen, Cat# SLF1000), and the LI-COR REVERT Total Protein Stain was used to ensure equal loading of sample as no housekeeping proteins are recommended for EVs [37]. For normalization of the NRP1 signal, the NRP1 band intensity was normalized using the intensity signal of the REVERT Total Protein Stain. To do so, the total signal intensity of each lane was calculated to determine the average total protein intensity of the entire blot; the intensity of each lane was then divided by the average intensity to determine the normalization factor for each lane. Each NRP1 band intensity was then divided by the normalization factor calculated for that lane to generate the NRP1/total protein stain (TPS) signal. The NRP1/TPS band intensity was transformed into % NRP1 abundance relative to the average band intensity of the hBM-MSC-sEV group. Briefly, each band intensity was divided by the average of hBM-MSC-sEV band intensity to generate an intensity ratio; the ratio was then converted to percentage (%) by multiplying the ratio by 100% to generate the % NPR1 abundance data.

### Transmission electron microscopy (TEM) analysis of hBM-MSC-sEVs

For TEM analysis, hBM-MSC-sEVs were isolated as described in the “hBM-MSC-sEV isolation” section from a starting volume of 15 mL of CCM. Following hBM-MSC-sEV isolation, the pellet was suspended in 300 μL of filtered PBS which was then filtered using vivaspin 300 kDa filters (Satorius, Cat#VS0651), following a rinse of the filters with 200 μL of filtered PBS centrifuged at 2000×*g* for 3 min. The concentrated hBM-MSC-sEVs were suspended in an equal volume of 4% PFA for 30 min. Next, two 50 μL drops of hBM-MSC-sEV/PFA suspension were deposited on parafilm and on which carbon-coated electron microscopy grids (Electron Microscopy Sciences, Cat#CF300-CU) were inverted and placed for 5 min on each 50 μL sample followed by a rinse with 50 μL drops of PBS on a sheet of parafilm. Grids were blotted on filter paper to remove excess and let dry for an hour before imaging. The TEM imaging was performed using a FEI Tecnai Spirit TEM with a LaB6 emitter, operating at 120 kV. The images were acquired with an Eagle camera with 4 k × 4 k resolution.

### Tandem mass tag (TMT) peptide labeling protocol for mass spectrometry (MS) based-proteomics analysis and statistical analysis

#### TMT peptide labeling protocol for MS

The hBM-MSC and hBM-MSC-sEV protein lysates from all five hBM donors were prepared according to the lysis protocol for mass spectrometry experiment described in the “hBM-MSC and hBM-MSC-sEV protein lysate preparation for Western blot and mass spectrometry analysis” section and quantified by BCA. The TMT protocol was followed according to the manufacturer’s protocol, with slight modifications. Refer to the TMT 10plex Mass Tag Labeling Kits and Reagents (Thermo Scientific, Cat#90113) for the product description. For more details on the protein lysate preparation and the TMT peptide labeling methods, refer to the Additional file 10. Briefly, the Orbitrap Fusion Tribrid Mass Spectrometer coupled to an Easy-nLC 1000 (Thermo Scientific) was used to analyze the multiplexed hBM-MSC (*n* = 5 donors) and hBM-MSC-sEV (*n* = 5 donors) TMT-labeled peptide samples (Additional file 8: Table S5). For more details on the LC-MS/MS analysis procedure, refer to the Additional file 10. Data processing was performed using the software package Proteome Discoverer 2.2 (Thermo Scientific). For more details, refer to the Additional file 10.

#### Statistical analysis of the MS-based proteomics and pathway enrichment analysis

Five thousand eighty-nine proteins were identified in the hBM-MSC dataset. Seven hundred seventy proteins were identified in the hBM-MSC-sEV dataset. After removing the proteins with 100% missing values in both of the datasets, 673 out of 770 proteins were compared in the differential analysis. The intensity data from the datasets was log_2_ transformed, and then the differential expression analysis between the hBM-MSC-sEV and hBM-MSC datasets was carried out using a paired *t* test for the 673 proteins. The *p* values were adjusted for multiplicity by a false discovery rate (FDR) controlling method based on the linear step-up method suggested by Benjamini and Hochberg [41]. Two hundred ninety-seven out of 673 proteins were revealed as statistically differentially enriched in hBM-MSC-sEV data (FDR *p* value < 0.05; ≥ 2.0 fold change), which are shown in the upper right section in the volcano plot. The statistical analyses were implemented by using SAS Enterprise Guide 5.1. When the 297 protein dataset was further analyzed by the Ingenuity Pathway Analysis (IPA) curated database for biological significance using the pathway enrichment tools, 270 proteins were mapped by IPA based on accession numbers and removal of duplicate IDs. The 270 protein dataset, herein called the 2-fold enriched hBM-MSC-sEV dataset, is the one presented in this study. For more details, refer to the Additional file 10.

## Results

### An optimized 7-day culture timeline of hBM-MSCs for EV production

**Fig. 1:**
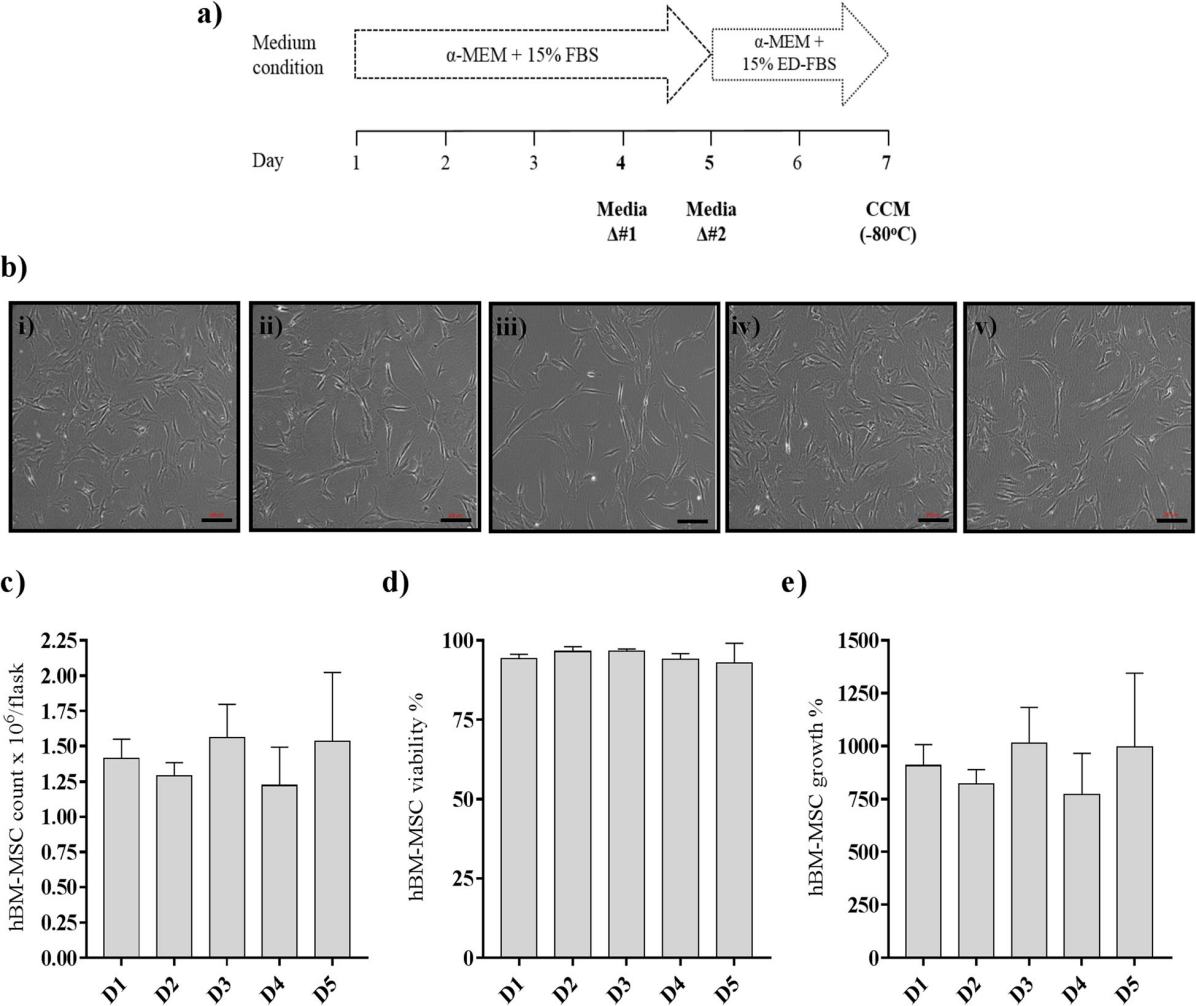
A 7-day culture timeline of hBM-MSCs designed to include a 48-h production time of sEVs. **a** A 7-day culture timeline of hBM-MSCs seeded at 1.4 × 10^5^ per T-175 flask at day #1 was established. The hBM-MSCs were grown under normal medium condition (α-MEM with 15% MSC-screened FBS) until day 5, where a medium change was included at day 4. At day 5, the culture medium was changed to an exosome-depleted medium (α-MEM with 15% exosome-depleted FBS) to perform a 48-h hBM-MSC-sEV production time. At day 7, the 48-h CCM produced under exosome-depleted FBS medium condition was collected and frozen at − 80 °C until ready for hBM-MSC-sEV isolation (ED: exosome depleted). **b** Representative phase contrast images at × 100 magnification taken for all five hBM-MSC donors (i–v) on day 7 showed typical MSC fibroblastic appearance (scale bar = 200 μm). Similar hBM-MSC **c** counts of viable cells (mean ± SD) per flask, **d** percentage viability (mean ± SD), and **e** growth percentage (mean ± SD) were measured. The data represent five hBM-MSC donors (*n* = 5 donors; D1-D5) analyzed in three independent trials (*n* = 3 experiments), each trial conducted with two technical replicates (*n* = 2 technical replicates)

hBM-MSCs derived from five young healthy male bone marrow donors (22–28 years old) at early passages (3 to 4) were characterized according to the ISCT’s minimal criteria for MSC characterization where plastic adherence was maintained, mesodermal differentiation (osteogenic and adipogenic) potential was confirmed, and surface marker expression for positive (≥ 95% for CD73, CD90, CD105) and negative (< 2% CD11b, CD19, CD34, CD45, CD79a, HLA-II; DR/DQ/DP) markers was validated by flow cytometry (Additional file 4: Table S1). hBM-MSC population doublings were also recorded at passages 1 and 2 by Texas A&M University Center, and again at passages 3 and 4 once working cell banks were generated in our laboratory (Additional file 4: Table S1). Comparable hBM-MSC growth and surface marker profile under our culture expansion conditions and the one of Texas A&M University Center were observed. Those qualified hBM-MSC preparations were used for small EV (sEV) production based on a 7-day culture timeline and a 48 h EV production (Fig. 1a and Additional file 1: Figure S1). Phase contrast images of all five hBM-MSC cultures confirmed a final overall confluence of ~ 70%, as well as showed a typical fibroblastoid appearance for MSCs (Fig. 1b). At the end of the 7-day timeline, the hBM-MSCs were harvested and live cell counts were recorded to later normalize EV counts per live cell as recommended per ISEV 2018 guidelines [37]. Similar live hBM-MSC counts (1.40 × 10^6^ ± 1.48 × 10^5^ mean ± SD viable cells) were obtained (Fig. 1c), viability over 95% was reached (Fig. 1d), and similar cell growth was attained for all five hBM-MSC donors (Fig. 1e), with no significant differences observed, showing consistency among all five hBM-MSC cultures. These results show that there were no observable phenotypic differences, which is important to overcome the variability of individual donor-derived cells for EV production.

### Size distribution analyses confirm the small EV category assignment

**Fig. 2:**
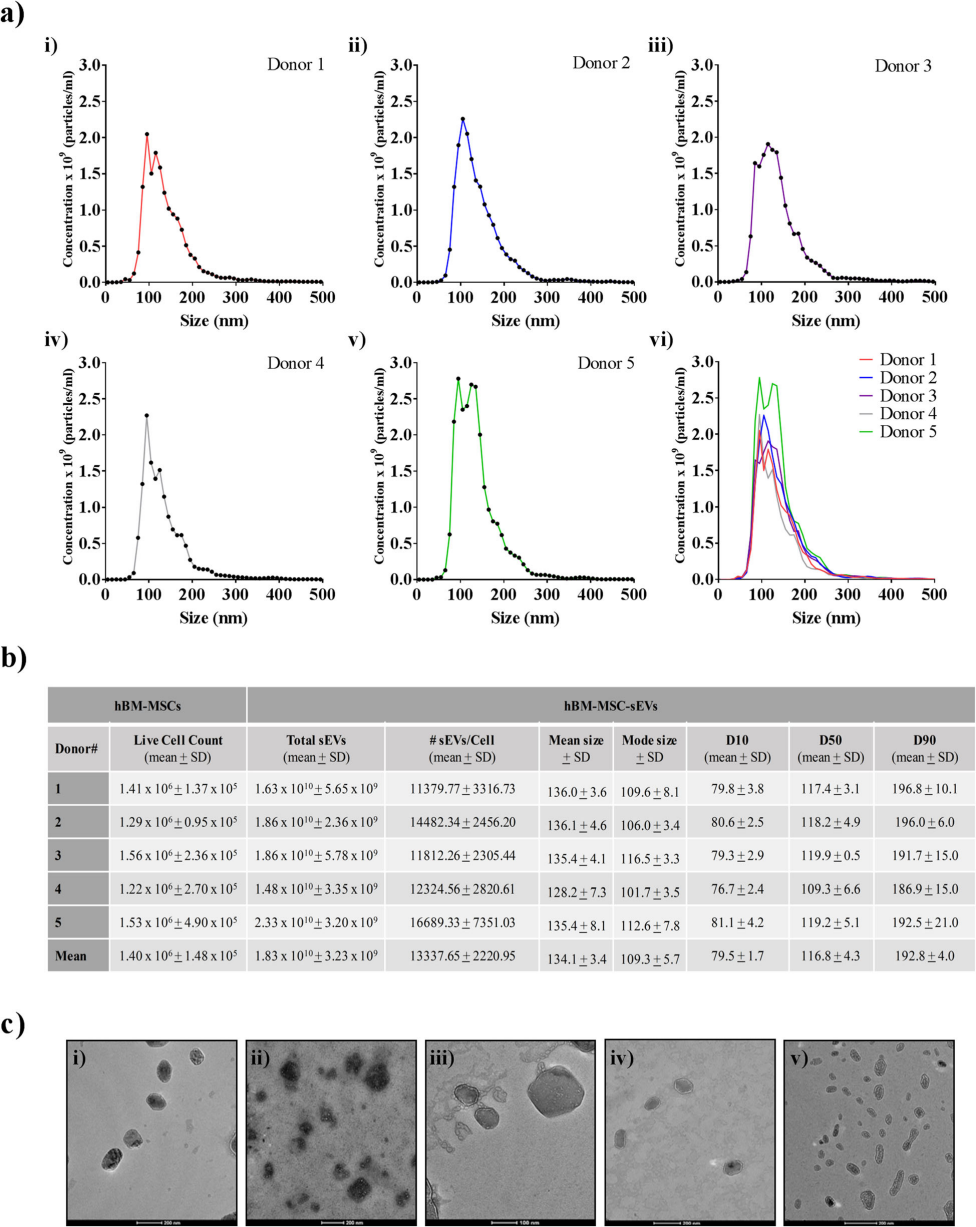
Size distribution analysis of hBM-MSC-sEVs performed with NTA and TEM confirmed small EV category assignment. **a** hBM-MSC-sEVs from (i) donor 1, (ii) donor 2, (iii) donor 3, (iv) donor 4, and (v) donor 5 were characterized by nanoparticle tracking analysis (NTA) as a means to estimate the hBM-MSC-sEV concentration and determine the EV size distribution. (vi) Superimposed NTA results of all five donors. *X*-axis designates size (nm) and *y*-axis designates concentration (mean) at different sizes of five individual healthy hBM-MSC donors (*n* = 5 donors). **b** Live cell counts of hBM-MSCs ± S. D, total mean concentration of hBM-MSC-sEV ± S. D, number of hBM-MSC-sEVs produced per hBM-MSC as a method of EV characterization, mean and mode size ± SD of hBM-MSC-sEVs, D10, D50, and D90 are shown as a method of nanoparticle size analysis. **c** Transmission electron microscopy (TEM) analysis confirmed the presence of hBM-MSC-sEV with the expected morphology, bilayer membrane, and size of small EVs (< 200 nm) (scale bars: D1, D2, D4, D5 = 200 nm, D3 = 100 nm). The data shown in **a** and **b** represent five hBM-MSC donors (*n* = 5 donors) analyzed in three independent trials (*n* = 3 experiments)

Single particle analysis techniques such as nanoparticle tracking analysis (NTA) are recommended by the ISEV 2018 minimal guidelines as one of the methods for the characterization of EVs with respect to both particle size and particle quantification [37]. Therefore, each of the five hBM-MSC-sEV samples produced over the 48 h period explained in Fig. 1 were quantified separately by NTA using a Nanosight NS300 instrument to determine the nanoparticle concentration and size distribution (Fig. 2a, b). Figure 2a shows similar size distributions for each of the five hBM-MSC-sEV samples where no differences were observed in the overall sEV concentration (1.83 × 10^10^ ± 3.23 × 10^9^ mean ± SD particles/mL), number of EV per live cell (13,338 ± 2221 mean ± SD particles/live cell), mean (134.1 ± 3.4 nm), and mode (109.3 ± 5.7 nm) particle size, which confirmed the small EV size category (50–200 nm) established by the ISEV 2018 guidelines [37] (Fig. 2b). Furthermore, transmission electron microscopy (TEM) corroborated the small EV size category showing nanoparticles ranging from 50 to 200 nm in diameter, as well as confirmed the presence of sEV with the expected lipid bilayer membranes among all five hBM-MSC donors (Fig. 2c).

### Flow cytometric and Western blot analyses confirm known small EV markers and low immunogenicity profile of hBM-MSC-EVs

**Fig. 3:**
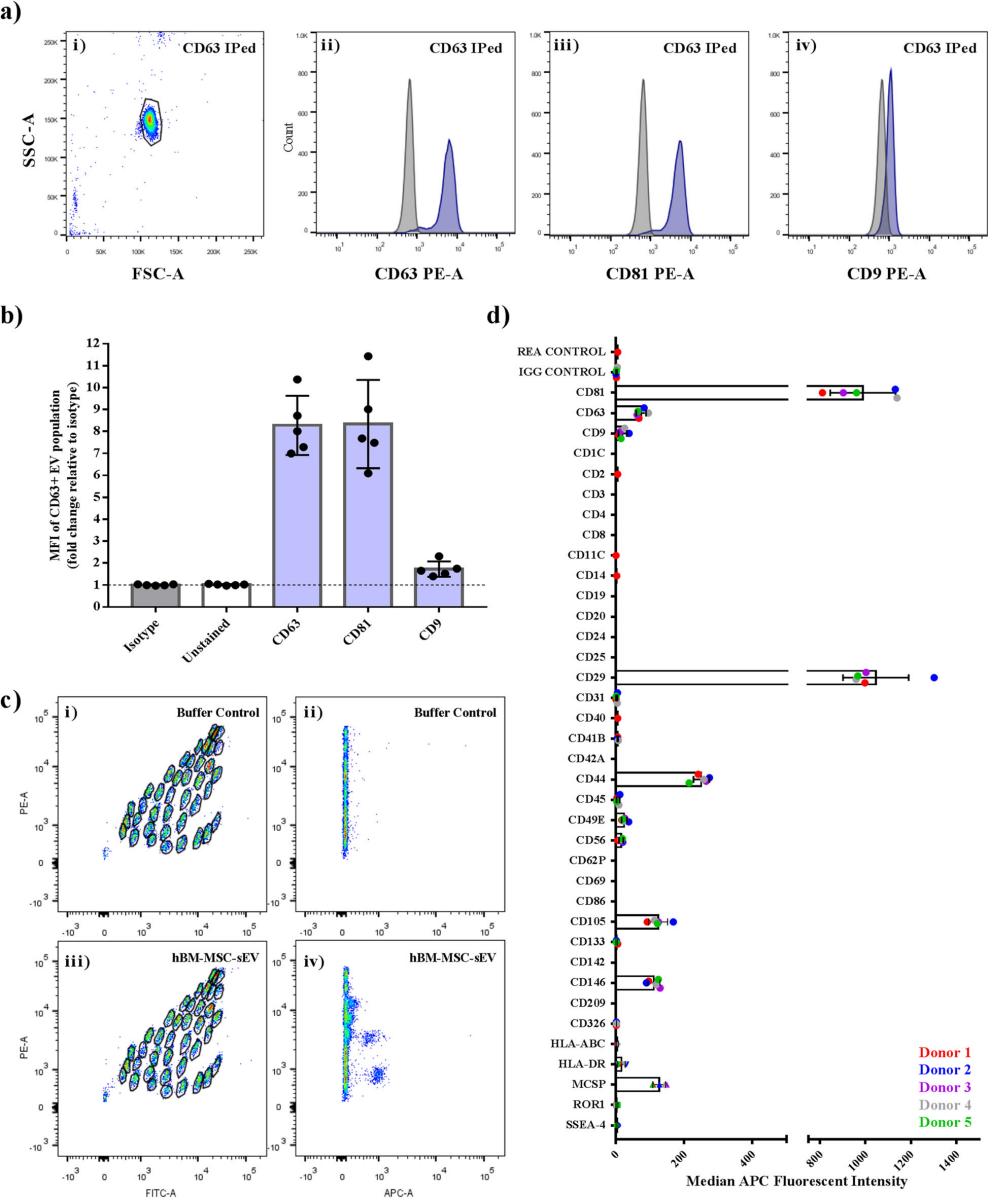
Flow cytometric analysis of CD9, CD63, and CD81 tetraspanin expression of the CD63+ hBM-MSC-sEVs population. **a** Representative flow cytometric plots of CD63 immuno-precipitated hBM-MSC-sEV population (i), counterstained for CD63 (ii), CD81 (iii), and CD9 (iv) (blue) against isotype control IgG1κ (gray) which served as a negative control. **b** Quantification of the median fluorescent intensity (MFI) data of the five hBM-MSC-sEV samples (*n* = 5 hBM-MSC donors) analyzed in three independent trials (*n* = 3 experiments). **c** Representative flow cytometric dot plots of hBM-MSC-sEV labeled MACSplex beads following CD63 detection. (i) Representative plots of buffer only negative control containing no nanoparticles (i and ii). Representative plots of hBM-MSC-sEV samples detected with CD63 counterstaining (iii and iv). **d** The IgG Isotype subtracted MFI data representative of five hBM-MSC-sEV samples (*n* = 5 hBM-MSC donors) analyzed in two independent trials (*n* = 2 experiments) displaying the relative surface abundance of various human CD markers

Characterization of sEVs requires the detection of proteins such as transmembrane or GPI-anchored proteins associated to plasma membrane and/or endosomes such as the tetraspanins CD63, CD81, and CD9. Therefore, CD63 antibody-coated 4 μm magnetic beads were used to immunoprecipitate the hBM-MSC-sEV CD63-positive population followed by counter staining of CD63, CD81, and CD9 epitopes to detect double positive populations. Isotype control IgG1κ served as a negative control for all three tetraspanins (Fig. 3a, b), and the median fluorescent intensities (MFI) for the isotype was used to calculate the fold change difference. High expression levels of CD63 (8.276 ± 0.604 fold change MFI) and CD81 (8.341 ± 0.899 fold change MFI) were measured on the CD63+ immuno-captured hBM-MSC-sEVs for all five hBM-MSC donors (Fig. 3b), whereas a highly variable low expression level was observed among the five donors for the CD9+ immuno-captured CD63+ hBM-MSC-sEV population (1.723 ± 0.1577 fold change MFI) as compared to the isotype (Fig. 3a, b). These results confirm that the common exosomal markers, CD63 and CD81, are highly detectable on hBM-MSC-EVs, but not CD9.

Next, an assessment of the general repertoire of 37 reported EV surface markers, which included tetraspanins CD63, CD81, and CD9 as well as an additional 34 markers, was conducted using a multiplex bead-based flow cytometric assay for further CD marker EV profiling (Fig. 3c, d). The results confirmed the high degree of expression of CD63 (74.2 ± 14.3 MFI ± SD) and CD81 (989.8 ± 143.8 MFI ± SD) on all five hBM-MSC-sEV samples, as well as a varying low expression of CD9 (19.8 ± 12.4 MFI ± SD). Additional strong positive surface markers were detected on all five hBM-MSC-sEV samples and included CD44 (250.4 ± 23.3 MFI ± SD), CD146 (111.4 ± 17.7 MFI ± SD), CD29 (1046.5 ± 144.5 MFI ± SD), MCSP (127.2 ± 18.8 MFI ± SD), and CD105 (123.9 ± 27.9 MFI ± SD) (Fig. 3c, d). One marker, CD49E, was detected consistently among all five hBM-MSC-EV samples although at an intermediate-positive expression level of 24.4 ± 7.9 MFI (Fig. 3c, d). Other EV markers were detected inconsistently among donors (present onto ≤ 2 hBM-MSC-sEV samples per marker) at a very low-positive APC fluorescence intensity levels (all at < 10 MFI) comprising CD45, CD31, CD56, CD41B, SSEA-4, ROR1, and HLA-DR and suggesting either a lack of expression (very close to background) or a very low expression linked to a particular hBM-MSC-sEV sample.

To further characterize the hBM-MSC-sEV samples produced, HSP90B1, a protein known to be associated with compartments other than plasma membrane or endosomes and described to be under represented or absent by the ISEV minimal guidelines of 2014 [38], was tested. The absence of HSP90B1 in all five hBM-MSC-sEV samples and its presence in all five hBM-MSC parental cultures was validated by Western blot and confirmed that our hBM-MSC-sEV preparation was not contaminated with HSP90B1 (Additional file 3: Figure S3a). Another confirmatory protein was tested by Western blot where the enrichment (or high abundance) of MMP-2 has been previously reported in sEV preparations [38]. Consistent with the EV literature, MMP-2 was detected in higher abundance in all five hBM-MSC-EV samples as compared to their corresponding parental hBM-MSC cultures (Additional file 3: Figure S3b). As EVs do not have a consistent housekeeping protein for normalization of protein load, we confirmed equal protein loading using a total blot stain (Additional file 3: Figure S3a-b).

### hBM-MSC-sEV proteomics dataset analyzed for biological significance reveals enriched membrane-associated proteins from the EV population

**Fig. 4:**
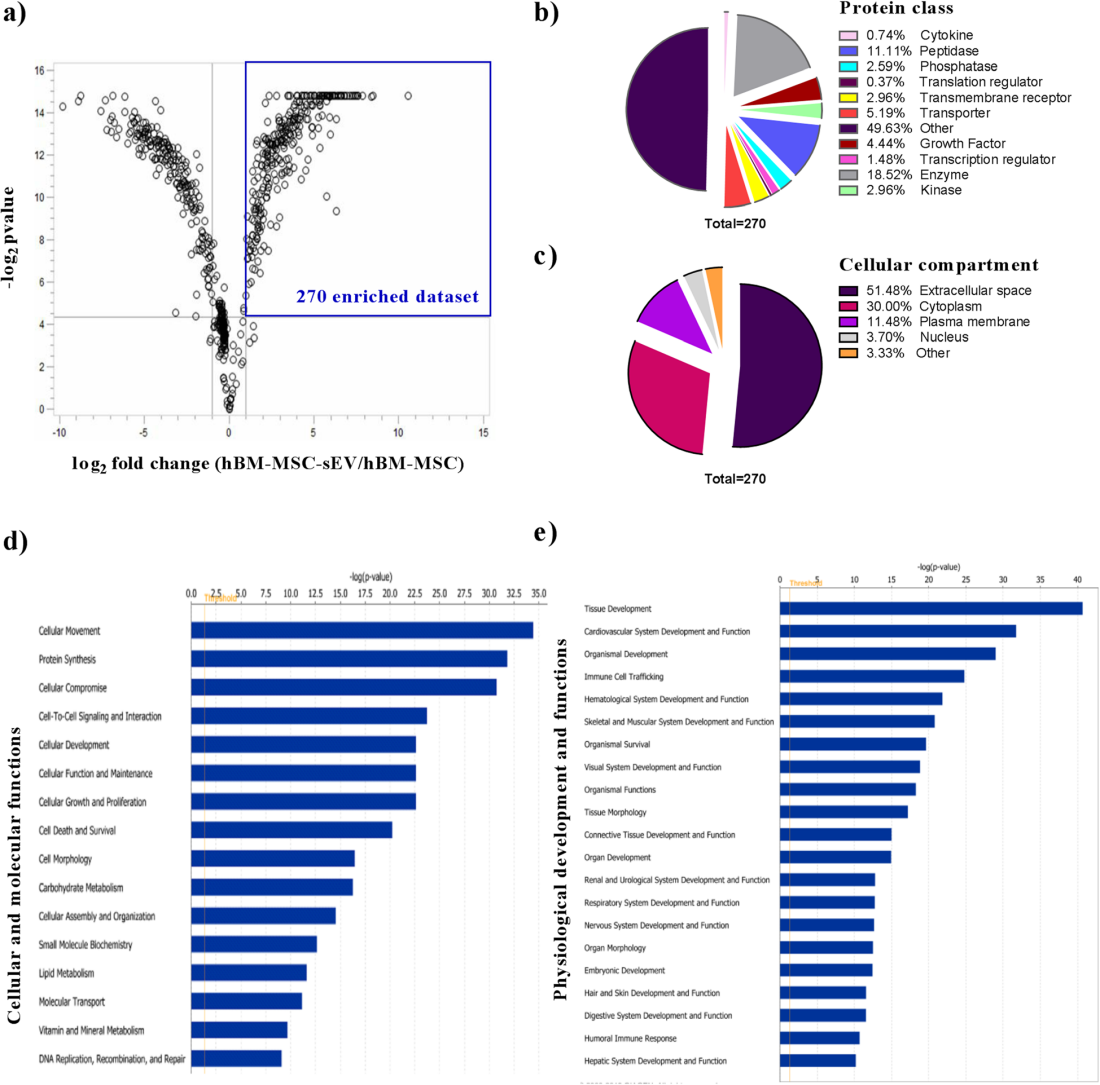
The twofold enriched hBM-MSC-sEV proteomics dataset analyzed for biological significance. Twofold enriched hBM-MSC-sEV protein dataset as compared to the parental hBM-MSCs. **a** Described in a volcano plot showing the significantly enriched proteins (upper right corner) in the overall proteomics dataset. **b** categorized into protein class, and **c** categorized into subcellular compartments using the Ingenuity Pathway Analysis (IPA) bioprofiler tool. The twofold enriched hBM-MSC-sEV protein dataset organized into **d** significantly upregulated cellular and molecular functions and **e** significantly upregulated physiological development and functions using IPA. *Y*-axis shows the significantly upregulated pathways and the *X*-axis shows the −log_10_ *p* value with a threshold set at 1.30 (−log_10_ *p* value) or *p* value of 0.05. Significantly upregulated pathways were calculated using a right-tailed Fisher’s exact test in IPA

After thorough phenotypical characterization and quality control assessment of hBM-MSC-sEVs, the comprehensive proteomic profile of sEVs and their parental hBM-MSCs from all five hBM-MSC donors was investigated using tandem mass tag (TMT)-labeled LC-MS/MS-based proteomics. Six hundred seventy-three proteins were commonly identified in hBM-MSC-sEVs, and their parental hBM-MSCs and analyzed for differential expression analysis. Out of the 673 proteins, 270 were enriched at least twofold changes (*p* value of < 0.05) in hBM-MSC-sEVs and mapped to the Ingenuity Pathway Analysis (IPA) database. These 270 represented the “2-fold enriched hBM-MSC-sEV dataset” and were further analyzed for functional significance using IPA (Fig. 4a and Additional file 5: Table S2). Using the bioprofiler tool in the IPA curated database, the “2-fold enriched hBM-MSC-sEV dataset” was classified into proteins class (Fig. 4b) and cellular compartment (Fig. 4c) categories. The enzymes (peptidase [11.11%], phosphatase [2.59%], enzyme [18.52%], kinase [2.96%]) dominated the protein class with a total percentage of 35.18% (Fig. 4b). Extracellular space proteins (51.48%) and cytoplasmic proteins (30.00%) represented the majority of cellular compartment proteins (Fig. 4c). When the “2-fold enriched hBM-MSC-sEV dataset” was organized into pathways related to two important biological categories, cellular and molecular functions as well as physiological development and functions, 16 and 20 significantly enriched pathways (right-tailed Fisher’s exact test of < 0.05) were found, respectively (Fig. 4d, e). The identities of the hBM-MSC-sEV proteins involved in regulating the top 2 most enriched pathways from each category (cell movement and protein synthesis for cellular and molecular functions; tissue development and cardiovascular system and function for physiological development and functions) are described in the Additional file 8: Tables S5a-d.

### Neuropilin 1 (NRP1) identified in hBM-MSC-sEVs

**Fig. 5:**
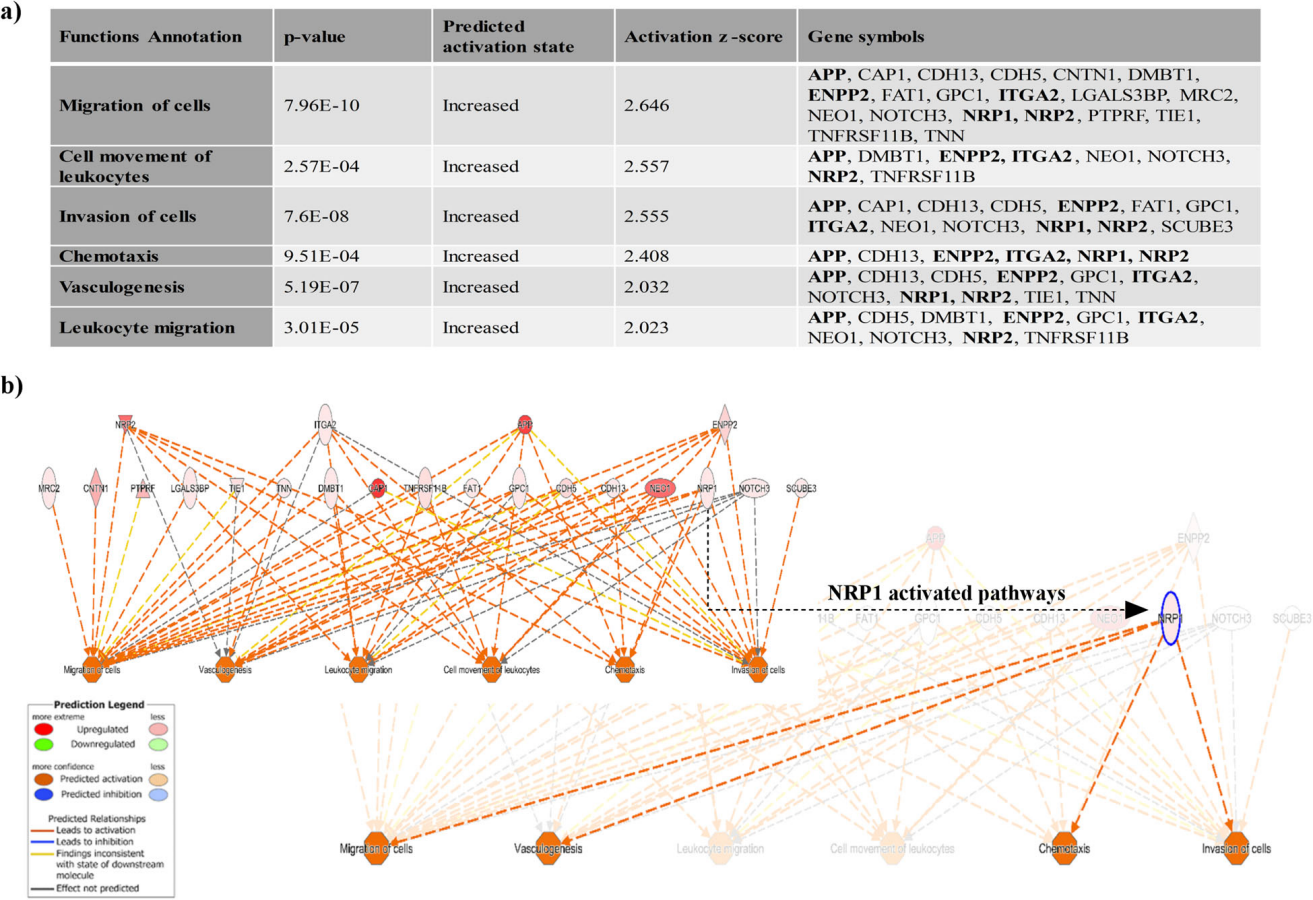
Functional pathway analysis of plasma membrane proteins from the twofold enriched dataset of hBM-MSC-sEVs. **a** Functional annotation of plasma membrane proteins from the twofold enriched dataset of hBM-MSC-sEVs showed an increase (activation *z*-score of ≥ + 2) in those categories: migration of cells, cell movement of leukocytes, invasion of cells, chemotaxis, vasculogenesis, and leukocyte migration. Shown in bold are the common proteins further inquired for the hierarchical network (**b**) of plasma membrane proteins from the twofold enriched dataset of hBM-MSC-sEVs highlighting the associated activated functions (activation *z*-score ≥ + 2), such as migration of cells, cell movement of leukocytes, invasion of cells, chemotaxis, vasculogenesis, and leukocyte migration with the NRP1 activated pathways highlighted

**Fig. 6:**
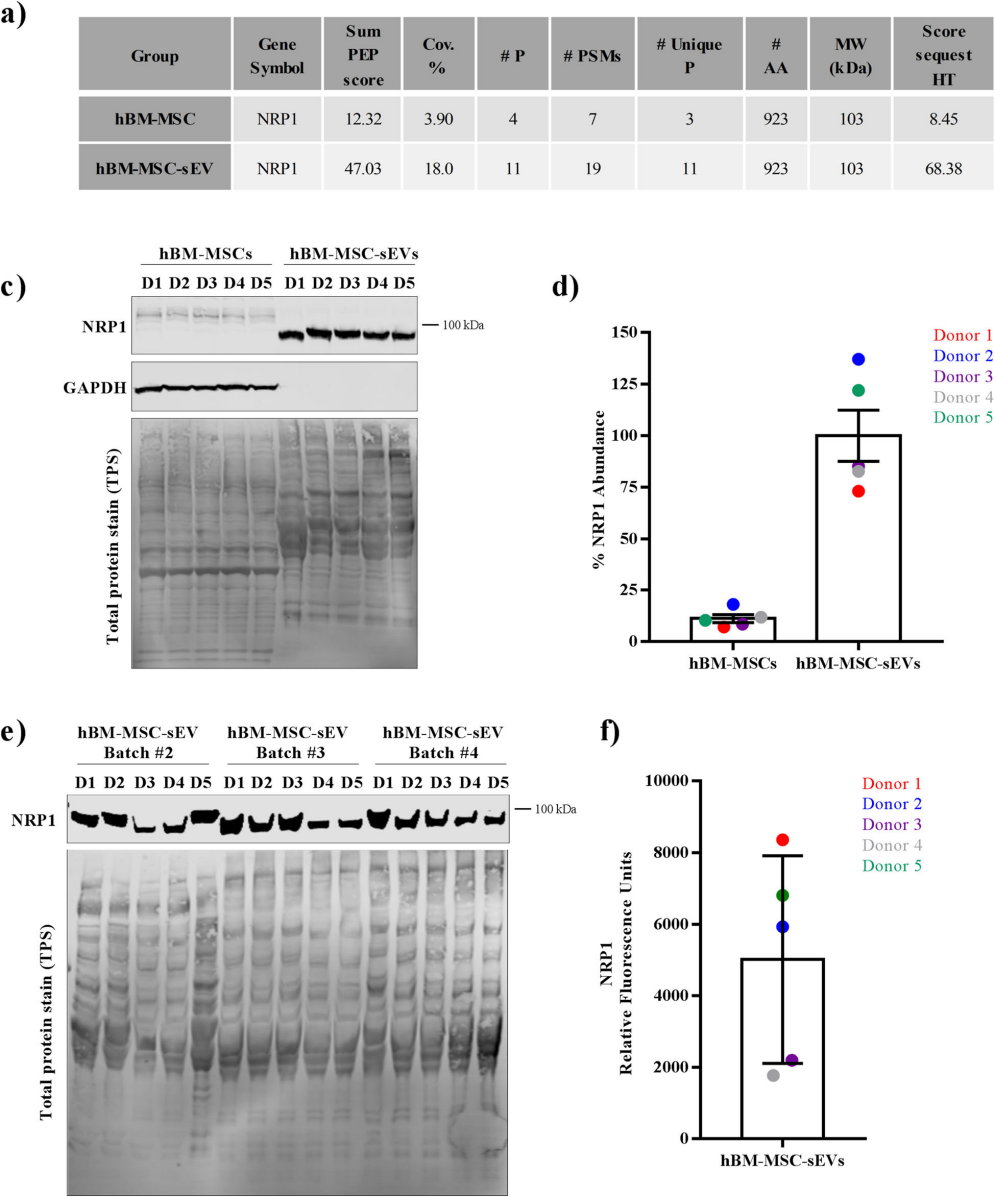
Validation of NRP1 selected from the twofold enriched hBM-MSC-sEV dataset by Western blot analysis. **a** Mass spectrometry-based proteomics data of NRP1 identified from the hBM-MSC and hBM-MSC-sEV groups that allowed its identification and quantification by LC-MS/MS. **b** Western blot validation of NRP1 mass spectrometry data showing the enrichment of NRP1 in hBM-MSC-sEVs as compared to hBM-MSCs from the samples analyzed by MS. **c** Quantification of % NRP1 abundance levels from the hBM-MSC and hBM-MSC-sEV groups obtained from **b** results. **d** Verification by Western blot of NRP1 from three additional EV production batches from all five hBM-MSC-sEV samples. **e** Quantification of NRP1 relative fluorescent units from the hBM-MSC-sEV samples from all five hBM-MSC donors. The data represent five hBM-MSC donors (*n* = 5 donors; donors 1–5) analyzed in two independent WB experiments from batch #1 EV production (**b**, **c**) or four independent WB experiments (**d**, **e**) from three additional separate EV production batches (batches #2–4)

In addition, our proteomic, flow cytometric, and Western blot analyses of the “2-fold enriched hBM-MSC-sEV dataset” verified 15 of the top 100 EV markers listed in the Exocarta database (http://www.exocarta.org/) confirming commonly found EV proteins from other studies and ours (Additional file 6: Table S3). As EV membrane proteins are an important aspect of EV activity and/or binding, we sought to identify EV-associated membrane proteins and found that 21 membrane-bound proteins were identified in the “2-fold enriched hBM-MSC-sEV dataset.” These 21 proteins were linked with processes that are predicted to be associated with an increase in migration of cells, cell movement of leukocytes, invasion of cells, chemotaxis, vasculogenesis, and leukocyte migration (Fig. 5). Among the proteins regulating these processes, NRP2, ITGA2, APP, and ENPP2 (shown in bold) (Fig. 5a) are common proteins involved all the functions, with NRP1 being involved in four out of the six activated pathways (migration of cells, invasion of cells, chemotaxis, and vasculogenesis) (Fig. 5b). LC-MS/MS-based proteomics data showed that NRP1 was identified based on 11 unique peptides with a protein sequence coverage of 18.0% from the hBM-MSC-sEV group, whereas the identification of NRP1 from the hBM-MSC group was based on 3 unique peptides and a sequence coverage of 3.9% (Fig. 6a, and Additional file 9: Table S6). When protein lysates generated for the LC-MS/MS experiment were analyzed for NRP1 abundance levels by Western blot from all five donors from both the hBM-MSC-sEV and the hBM-MSC groups, similar results were obtained where an enrichment in NRP1 abundance levels were found in hBM-MSC-sEVs, which corroborated the mass spectrometry data (Fig. 6b, c). To ensure reproducibility and reliability in the detection of NRP1 as a hBM-MSC-sEV protein surface marker, three additional separate batches of hBM-MSC-sEV lysates from all five hBM-MSC donors were produced and analyzed for the presence of NRP1. Results obtained for these additional three rounds of experiments showed comparable results whereby the abundance levels of NRP1 were also detectable at a high degree in all five hBM-MSC-sEV samples from all three independent batches (Fig. 6d, e).

## Discussion

The safe and efficacious use of EV-based therapeutics will rely on the identification of their molecular components, which will serve to determine their quality, identity, safety, and potency attributes. This was demonstrated in a human clinical trial where the hMSC-sEV sample used for treating a patient with steroid-refractory acute GvHD was selected based on a higher ratio of anti-(IL-10) to pro-(IFNγ) inflammatory cytokine content among four EV samples derived from different unrelated bone marrow donors [25]. The clinical GvHD symptoms improved significantly shortly after the start of the MSC-exosome/sEV therapy where the diarrhea volume was reduced and the cutaneous and mucosal GvHD showed a remarkable response within 2 weeks following therapy. However, long-term safety assessment of the sEV treatment was not possible since the patient died of pneumonia 7 months post-MSC-exosome/sEV therapy. Owing to the clinical response and the fact that the patient was stable for several months, the authors concluded that the MSC-derived exosomes/sEVs may provide a potential new and safe tool to treat therapy-refractory GvHD [25]. There is accumulating evidence that the surface protein marker signature and the overall protein constitution reflect the state in which the parental cell type is when releasing the EVs [4]. In many cases, the presence of specific cargo molecules has been linked to the specific EV functions. For example in the context of cancer, specific EV cargo has been shown to promote pro-tumorigenic effects, shape the tumor microenvironment, and enhance metastasis [27]. The parental cell activation state can therefore influence the molecular composition, thereby dictating the therapeutic activity of EVs [28]. Based on this characteristic, sEVs or exosomes are regarded as predictive or prognostic biomarkers of disease, more particularly in cancer [42]. Identifying proteins, particularly surface markers, is therefore vital to qualify, identify, and ensure safety of EV-based therapeutics.

Using a multiplex bead-based flow cytometric assay as a targeted approach to detect the expression of 37 reported EV surface markers and to qualify our hBM-MSC-sEVs, we identified high expression levels for CD63, CD81, CD44, CD146, CD29, MCSP, and CD105 among all five hBM-MSC-EVs. CD9 and CD49E depicted a lower varying expression levels, although consistent among all five donors. Similarly, Wiklander and colleagues recently also reported the detection of CD63, CD81, CD44, CD29, MCSP, CD105, and CD49E from human Tert+ immortalized BM-MSCs, but not CD146 [43]. We found that the purported EV/exosome marker CD9 was varying in expression among all five hBM-MSC-sEV samples, all being detectable at a very low level, which is supported by the study of Wiklander and colleagues where no CD9 detection was reported from immortalized MSCs [43]. Based on our flow cytometric results, CD63 is considered to be a more suitable common EV marker for hBM-MSC-sEVs. Indeed, we detected a strong CD63 signal on all five hBM-MSC-sEV samples using two complementary CD63 bead-based flow cytometric assays (the multiplex bead-based and the CD63-coated 4 μm bead flow cytometric assays) where both were based on the immunoprecipitation of CD63 + sEVs and counterstaining using either CD63-PE or CD63-APC conjugates. Similarly, CD81 was strongly detectable using these two bead-based flow cytometric approaches, where both the CD63+ immunoprecipitated sEVs showed strong positivity for CD81, and the CD81+ immunoprecipitated EVs showed positivity for CD63. Besides the broadly detected common CD63 and CD8 tetraspanins, where their presence is expected due to the biogenesis of EVs, MSC-characteristic positive markers (CD44, CD146, and CD105) were also identified on hBM-MSC-sEVs. The presence of MSC-positive CD44, CD146, and CD105 markers provide further evidence that the surface marker profile of sEVs reflects the one of the parental cell. On the other hand, the presence of MCSP on hBM-MSC-sEVs and its contributions is less understood, although its presence on hBM-MSCs has been previously reported [44].

To identify novel surface markers that could serve to qualify and identify hBM-MSC-sEVs, we used a non-targeted approach based on mass spectrometry and identified 21 membrane or membrane-bound proteins enriched in hBM-MSC-EVs. Among the MSC biologically relevant membrane proteins identified, neuropilin 1 (NRP1) was selected for complementary validation by Western blotting based on its high mass spectrometry quality results and its relevant biological functions for hMSCs. To assess NRP1’s effectiveness as a potential identity marker for MSC-sEVs, three additional sEV batches were processed independently for all five hBM-MSC donors and confirmed NRP1 as a reliable marker for hBM-MSC-EVs as its presence was consistency detected. NRP1 is a membrane-associated glycoprotein containing a small cytoplasmic domain and multiple extracellular domains, which complexes with both VEGFR-1 and VEGFR-2 and potentiates the binding of VEGF-A [45, 46]. In a cell, the primary functions of NRP1 are vascular and neural development. NRP1 is a co-receptor for VEGF and semaphorin and interacts selectively with different members of the VEGF and semaphorin families [45] where it potentiates cell motility in relation to proangiogenic activity and neural development [45]. NRP1 also regulates platelet-derived growth factor receptors (PDGFR) and NRP-1/PDGFR cross-talk is essential for vascular remodeling [47]. The association of NRP1 with vasculogenesis was observed in our bioinformatics approach where the presence of NRP1 was predicted to activate this function. A functional linkage of NRP1-containing sEVs and vasculogenesis has never been reported, although the angiogenic potency of MSC-EVs containing angiogenic factors, such as NRP1, has been discussed in a recent study conducted by Eirin and colleagues [48]. The authors showed that MSC-EVs promoted angiogenesis and vascular repair in two chronic post-stenotic kidney experimental models (MetS and RVD) and identified NRP1 by mass spectrometry as one of the angiogenic cargo constituents of MSC-sEVs [48]. The observations suggesting that sEVs can promote beneficial angiogenesis through their selectively enriched cargo are also true for tumor angiogenesis. It has been reported that the delivery of NRP1 via exosomes/sEVs from MDCK oncogenic cells to the surface of recipient endothelial cells leads to increased responsiveness to soluble angiogenic ligands such as VEGF-A, thereby activating tumor angiogenesis [28]. This transfer and activation of NRP1 via sEVs from oncogenic cells to recipient endothelial cells may alter endothelial cell surface interactions and increase their responsiveness to soluble angiogenic ligands in the tumor microenvironment [28].

Our work confirms the identification of NRP1 on hBM-MSC-sEVs by mass spectrometry as well as its verification by Western blotting from five different MSC donors at low passages (#3 to 4). Supporting our NRP1 results, Yuan and colleagues identified NRP1 among 701 mitogenic EV proteins by mass spectrometry from primed MSCs (1% oxygen tension for 48 h) [39], although not further validated by a complementary mean of verification such as Western blotting, and identified from only three MSC donors at higher passages (#6). These results further strengthen the importance of pursuing the validation of NRP1 as a MSC-EV biomarker to understand its role in the therapeutics context of EV therapy.

## Conclusion

A thorough phenotypic and proteomic characterization of EVs derived from five healthy male hBM-MSC donors at low passage (#3 to 4) was conducted where the qualified EVs fit under the small EV classification based on the ISEV 2018 guidelines. The qualified hBM-MSC-sEV samples subjected to MS-based proteomics analysis yielded important potential membrane surface receptor candidates among which NRP1 was identified and validated by Western blotting and found to be consistently expressed on hBM-MSC-EVs from five hBM-MSC donors. This study will provide new insights into the underlying molecular mechanism of action of hBM-MSC-EVs via proteins, which can be translated to development of EV potency and product marker-based assays upon further investigations.

## Supplementary information

**Additional file 1: Figure S1.** Schematic representation of the hBM-MSC-sEV isolation processing workflow. A 7-day timeline was designed for hBM-MSC-sEV production where the cell-conditioned medium was collected and processed for EV isolation as described.

**Additional file 2: Figure S2.** Analysis by flow cytometry of hBM-MSC positive (CD73, CD90 and CD105) and negative (CD34, CD45, CD11B, CD19 and HLA-DR) surface marker expressions set by the ISCT’s minimal criteria for MSC characterization.

**Additional file 3: Figure S3.** Western blot analyses comparing HSP90B1 and MMP2 protein expression levels for hBM-MSC-sEV and hBM-MSC protein lysates. Reverse expression pattern was found for hBM-MSC-sEVs, where negative (HSP90B1) and positive (MMP2) expression was detected for hBM-MSC-sEV as compared to hBM-MSCs.

**Additional file 4: Table S1.** hBM-MSC characterization for surface identity markers, differentiation potential to mesodermal lineage and population doublings.

**Additional file 5: Table S2.** TMT labelled MS-based proteomic analysis of the hBM-MSC-EV 2-fold enriched dataset.

**Additional file 6: Table S3.** EV proteins of the top 100 Exocarta database identified in the 270 protein hBM-MSC-EV dataset.

**Additional file 7: Table S4.** TMT labels used for the mass spectrometry analysis of the hBM-MSCs and hBM-MSC-EV samples.

**Additional file 8: Table S5. (a)** The most enriched pathway for the analysis of the cellular and molecular biological category, **(b)** The second most enriched pathway for the analysis of the cellular and molecular biological category, **(c)** The most enriched pathway for the physiological development and functions biological category, **(d)** The second most enriched pathway for the physiological development and functions biological category.

**Additional file 9: Table S6.** Peptide groups for Neuropilin-1 (accession Q68DN3) detailing the 11 peptides identified with the SequestHT algorithm in Proteome Discoverer 2.2.

**Additional file 10:** Additional Material and Methods

## Data Availability

The proteomics dataset generated from mass spectrometry and analyzed during the current study is available in the Additional section.

## Abbreviations

BM: Bone marrow
CCM: Cell-conditioned medium
ED: Exosome-depleted
EV: Extracellular vesicle
GvHD: Graft-versus-host disease
hMSC: Human mesenchymal stromal/stem cells
hBM-MSC: Human bone marrow-derived mesenchymal stromal/stem cells
HLA: Human leukocyte antigen
IFN-γ: Interferon gamma
IL-10: Interleukin 10
IPA: Ingenuity Pathway Analysis
ISCT: International Society for Cellular Therapy
ISEV: International Society for Extracellular Vesicle
LC-MS/MS: Liquid chromatography tandem mass spectrometry
MDCK: Madin-Darby Canine Kidney
MetS: Metabolic syndrome
MFI: Median fluorescence intensity
MHC: Major histocompatibility complex
MS: Mass spectrometry
MSC: Mesenchymal stromal/stem cell
NTA: Nanoparticle tracking analysis
NRP1: Neuropilin 1
PDGFR: Platelet-derived growth factor receptor
PEG: Polyethylene glycol
PFA: Paraformaldehyde
RIPA: Radioimmunoprecipitation assay
RVD: Renovascular disease
SEC: Size exclusion chromatography
sEV: Small extracellular vesicle
TEAB: Triethylammonium bicarbonate
TEM: Transmission electron microscopy
TFF: Tangential flow filtration
VEGF-A: Vascular endothelial growth factor-A
VEGFR-2: Vascular endothelial growth factor receptor-2
