# Supplementary figures and images for: A comprehensive proteomics profiling identifies NRP1 as a novel identity marker of human bone marrow mesenchymal stromal cell-derived small extracellular vesicles
Source: Stem Cell Res Ther. 2019 Dec 18;10:401. doi: 10.1186/s13287-019-1516-2 (PMC6921509; doi:10.1186/s13287-019-1516-2)

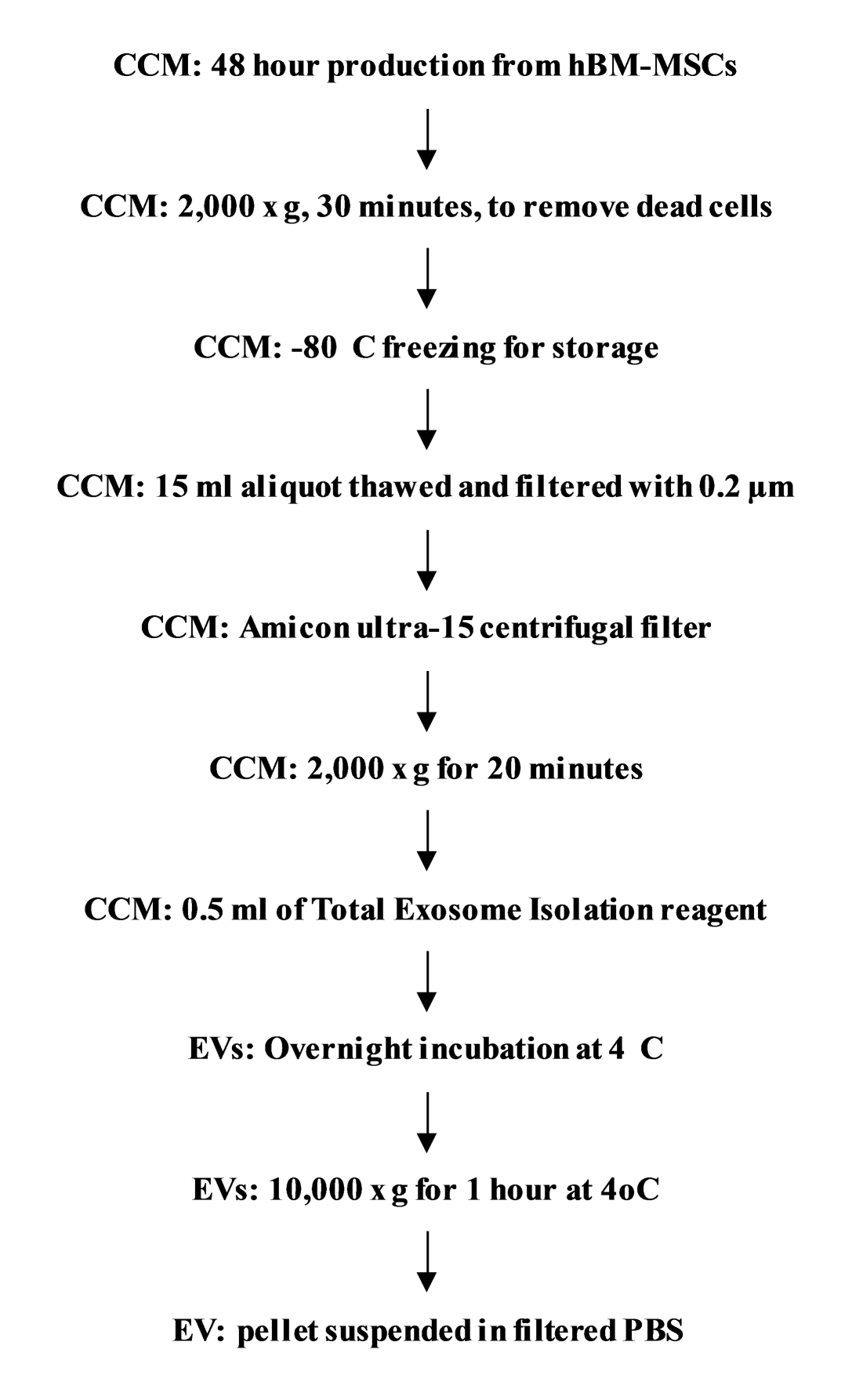

Supplement: Supplementary file 1 — Additional file 1: Figure S1. Schematic representation of the hBM-MSC-sEV isolation processing workflow. A 7-day timeline was designed for hBM-MSC-sEV production where the cell-conditioned medium was collected and processed for EV isolation as described. [file 13287_2019_1516_MOESM1_ESM.tif]

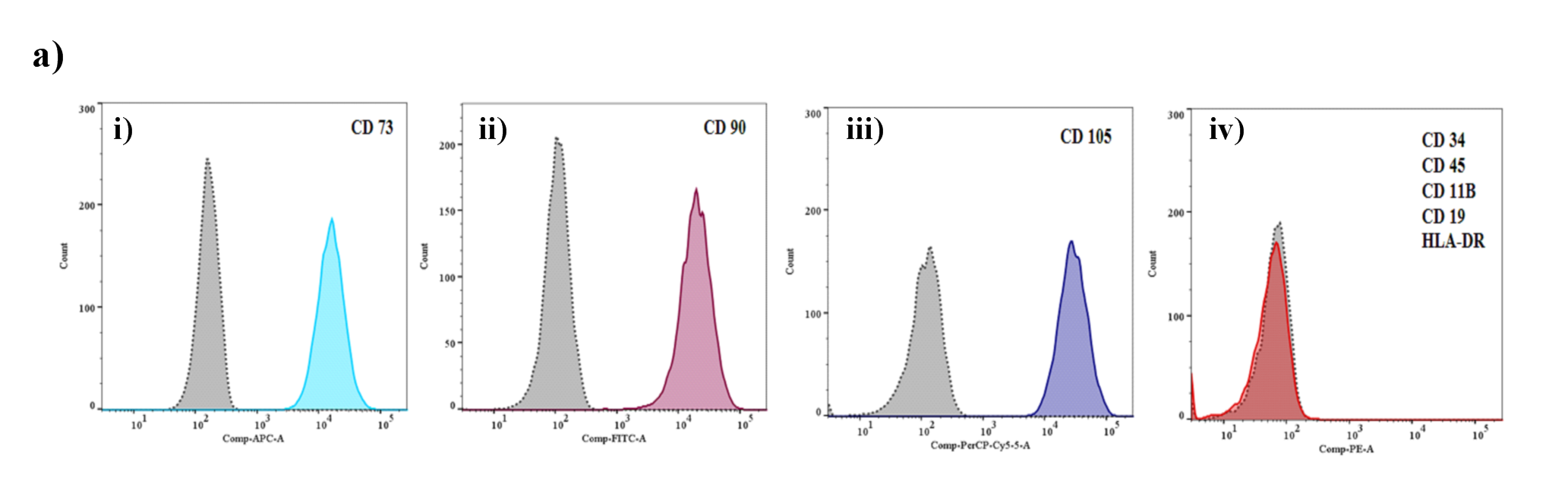

Supplement: Supplementary file 2 — Additional file 2: Figure S2. Analysis by flow cytometry of hBM-MSC positive (CD73, CD90 and CD105) and negative (CD34, CD45, CD11B, CD19 and HLA-DR) surface marker expressions set by the ISCT’s minimal criteria for MSC characterization. [file 13287_2019_1516_MOESM2_ESM.tif]

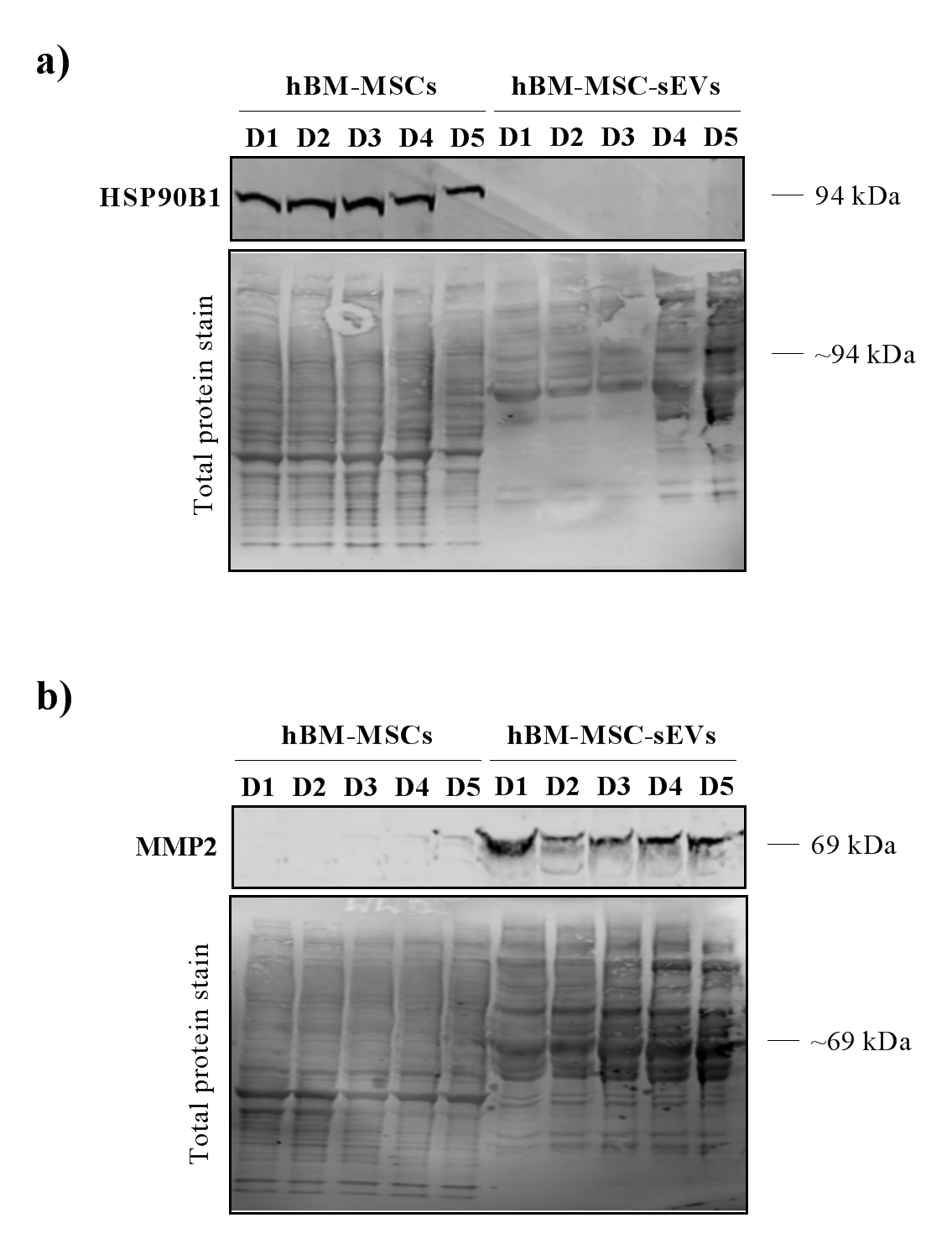

Supplement: Supplementary file 3 — Additional file 3: Figure S3. Western blot analyses comparing HSP90B1 and MMP2 protein expression levels for hBM-MSC-sEV and hBM-MSC protein lysates. Reverse expression pattern was found for hBM-MSC-sEVs, where negative (HSP90B1) and positive (MMP2) expression was detected for hBM-MSC-sEV as compared to hBM-MSCs. [file 13287_2019_1516_MOESM3_ESM.tif]
