# Supplementary material for: A comprehensive proteomics profiling identifies NRP1 as a novel identity marker of human bone marrow mesenchymal stromal cell-derived small extracellular vesicles
Source: Stem Cell Res Ther. 2019 Dec 18;10:401. doi: 10.1186/s13287-019-1516-2 (PMC6921509; doi:10.1186/s13287-019-1516-2)
Supplement: Supplementary file 10 — Additional file 10: Additional Material and Methods [file 13287_2019_1516_MOESM10_ESM.docx]

**Additional Material and Methods**

**1.0 Tandem mass tag (TMT) labeling protocol for mass spectrometry (MS) based‑proteomics analysis.**

**1.1 Protein sample extract procedure**

100 µg of hBM-MSC (n=5) and hBM-MSC-sEV (n=5) protein lysates were transferred into a new microcentrifuge tube and adjusted to a final volume of 100µl with 100mM TEAB. 5µl of the 200mM TCEP solution was added to the 100µg/100µl protein sample and incubated at 55°C for 1 hour in a heating block. One tube of iodoacetamide (9mg) was dissolved in 132µl of 100mM TEAB to make a 375mM iodoacetamide solution. 5µl of the 375mM iodoacetamide solution was then added to each of the 100µg protein samples and incubated for 30 minutes while being protected from light at room temperature. Next, a three stepwise acetone precipitation procedure was performed by adding pre-chilled (-20°C) acetone (4 times the volume of 100µg protein added 3 times every 15 minutes). Samples were then kept at -20°C for 90 minutes, while vortexing every 15 minutes after which the samples were precipitated overnight at -20°C. The next morning, samples were centrifuged at 14,000g for 10 minutes at 4°C to remove the acetone, which was done carefully without dislodging the protein pellet. The pellet was then air dried for ~10 minutes.

**1.2 Protein digestion preparation**

100µg of the acetone-precipitated protein pellet was suspended with 100µl of 100mM TEAB (flick, slow vortex to dissolve). Immediately before use, 20µl of the trypsin storage solution was added to the bottom of the trypsin glass vial from the TMT 10plex Mass Tag Labeling Kits and Reagents (Thermo Scientific, Cat#90113) and incubated for 5 minutes. Then 2.5µl of trypsin (i.e., 2.5µg) was added per 100µg of protein sample and incubated overnight at 37°C in a Thermomixer C at 500 rpm.

**1.3 Protein labeling procedure using TMT labels**

The next morning, protein digest concentration was measured using the Thermo Scientific Pierce Quantitative Colorimetric Peptide Assay kit (Thermo Scientific, Cat#23275) according to the manufacturer’s protocol. Two technical replicates of each donor were labelled with TMT reagents on two different days. TMT label reagents were first equilibrated at room temperature immediately before use and 41µl of anhydrous acetonitrile was added to each TMT label vial (0.8mg) which was then allowed to dissolve for 5 minutes with occasional vortexing. 41µl of the reconstituted TMT label reagent was added to each of the 100µl samples and incubated for 1 hour at room temperature. Next, 8µl of 5% hydroxylamine was added to each sample and incubated for 15 minutes to quench the reaction. Samples were stored at -80°C until ready for analysis.

**1.4 Samples for mass spectrometry analysis**

Samples were thawed on ice, 10 µg (in 15 µl) of each of the 2 technical replicates of the 5 hBM-MSC samples were combined, and separately 10 µg (in 15 µl) of each of the 2 technical replicates of the 5 hBM-MSC-sEV samples were combined and these two mixtures were each lyophilized, dissolved in 300µl 0.1% trifluoroacetic acid (TFA) and fractionated. For the high pH-reversed phase fractionation method, spin columns from Pierce (Cat#84868) were conditioned and used according to the manufacturer’s instructions. Fractions were eluted in 300µl of each TMT‑labeled peptide elution solution as follows:


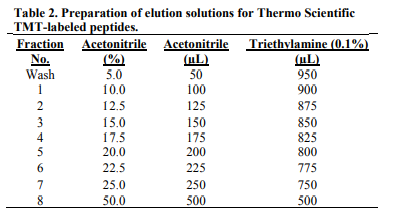


Fractions were evaporated to dryness and suspended in 20µl injection buffer (0.1% formic acid in water) prior to LC–MS/MS analysis.

**1.5 LC-MS/MS analysis**

The Orbitrap Fusion Tribrid Mass Spectrometer coupled to an Easy-nLC 1000 (Thermo Scientific) was used to analyse the TMT labeled peptides. The instrument was calibrated by infusion prior to analysis with a mixture of caffeine, MRFA, and Ultramark 1621. For each fraction 6 µl were analyzed by loading onto a NanoViper Acclaim pepmap 100 trap column (75 µm 20 mm with 3 µm beads) and desalting with 0.1% formic acid in water (solvent A), before separating on a NanoViper Acclaim pepmap RSLC C18 reverse-phase analytical column (75 µm 150 mm with 3 µm beads). Chromatographic separation was achieved at a flow rate of 0.300 µl/min over 140 min in seven linear steps as follows (solvent B was 0.1% formic acid in acetonitrile): initial, 5% B; 3 min, 5% B; 5 min, 10% B: 110 min, 25% B; 130 min, 60% B; 135 min, 90% B; 140 min, 90% B. The eluting peptides were analyzed in data-dependent mode for both MS2 and MS3 methods. A MS survey scan of 400−1500 m/z was performed in the Orbitrap at a resolution of 120000 and an AGC target of 4 × 105. The top speed mode was used to select ions for MS2 analysis, requiring charge state 2-7 and dynamic exclusion 40 s with a ±10 ppm window, and monoisotopic precursor selection. During the MS2 analyses, precursors were fragmented by HCD at collision energy of 35% +/- 10%, followed by Turbo IonTrap analysis using automatic m/z normal scan range. Precursors were isolated in the quadrupole using a width of 1.2, accumulated to an AGC target of 1 × 104 or a maximum injection time of 50ms. The 10 most intense fragments in MS2 spectra were selected for MS3 analysis with the filters mass range 400-1200, precursor exclusion +/- 5 m/z, and TMT isobaric tag loss exclusion. MS3 analysis was performed in the Orbitrap at resolution 60000 from 100-500 m/z, precursors isolated using a 2 m/z isolation window, accumulated to an AGC target of 5 × 104 or a maximum injection time of 120 ms. The MS3 precursor population was isolated using the SPS waveform and then fragmented by HCD, with a normalized collision energy set to 65.

**1.6 LC-MS/MS data processing**

The software package Proteome Discoverer 2.2 (Thermo) was used to process the data. The SEQUESTHT algorithm was used to search MS2 spectra against a database containing human proteins (Uniprot-Trembl, downloaded 20171116, 124124 entries) concatenated with a database of common contaminants (cRAP). Database search parameters were 10 ppm precursor ion tolerance, 0.6 Da fragment ion tolerance, and allowing up to two missed cleavages. Fixed modifications were TMT tags on peptide N termini/lysine residues (+229.162932 Da) and carbamidomethylation of cysteine residues (+57.02146 Da) while variable modifications were N-terminal acetylation (+42.011 Da), methionine oxidation (+15.99492 Da).. An MS2 spectra assignment false discovery rate (FDR) of less than 1% was achieved by applying Percolator algorithm. For quantification, a 20 ppm integration tolerance with the most confident centroid integration method was used. To account for differences in sample handling samples were normalized on total peptide amount. Missing values were replaced with minimum values, and only MS3 spectra having minimum average signal to noise (S/N) ratio of 10 were accepted for quantification.

**1.7 Statistical analysis of the mass spectrometry-based proteomics and pathway enrichment analysis**

For each protein identified by mass spectrometry, there were five observations related to the protein intensity data of the hBM-MSC samples (i.e. one observation from each of the five hBM donors), the same is true for the hBM-MSC-sEV samples (i.e. five hBM donors). In total, 5089 proteins were identified in the hBM-MSC dataset and 770 proteins were identified in the hBM‑MSC‑sEV dataset. After removing the proteins with 100% missing values in both of the datasets, 673 out of 770 proteins was compared in the differential analysis. While a missing value of a protein in the hBM-MSC-sEV was imputed by the protein specific minimal value (PSMV) across both datasets, the absolute minimal value (AMV), which is 4.7, from the hBM-MSC-sEV dataset was used to impute all the missing values in the hBM-MSC dataset. Given the “true” value of a missing value is unknown, the advantage of the AMV approach for the missing value imputation in hBM-MSC data is that it will less likely mistakenly label a protein as not enriched in hBM-MSC-sEV. For example, when the “true” value of a protein was closed to AMV, if the PSMV approach was used, it would unreasonably lead to smaller fold change (sEV vs. MSC) and larger p-value for the comparison of this protein; consequently, the protein was more likely to be categorized as not enriched in hBM-MSC-sEV. Therefore, AMV approach is considered as an adequate approach for the missing value imputation in hBM-MSC dataset. The above‑mentioned data imputation approach was adopted based on the consideration of trying to avoid prematurely identifying a protein as not differentially expressed. The intensity data of both datasets were log_2_‑transformed after the imputation. The transformed values of each of 673 proteins of hBM‑MSC‑sEVs and of hBM-MSCs were compared using a paired t‑test procedure. The p-values were adjusted for multiplicity by a false discovery rate (FDR) controlling method based on the linear step-up method suggested by Benjamini and Hochberg (1995) (1). 297 proteins were considered significantly up-regulated in hBM‑MSC‑sEV dataset (vs. hBM-MSC dataset) based on adjusted FDR p-values of <0.05 and a cut-off point of at-least-2 fold changes, and were further analyzed. There are different definitions of fold changes and the one used in our analysis is the ratio between arithmetic means. The volcano plot was generated from the 673 protein dataset found in common between both hBM-MSC-sEVs and hBM-MSCs, and the statistically (adjusted p-values of <0.05) differentially (≥ 2.0 fold change) regulated proteins are underscored by the upper left and right sections; 297 proteins enriched in hBM-MSC-sEV data is presented in the upper right section in the plot. The statistical analysis was accomplished by using SAS Enterprise Guide 5.1. When the statistically differentially upregulated 297 protein dataset was further analyzed by the Ingenuity Pathway Analysis (IPA) curated database for biological significance using the pathway enrichment tools, 270 proteins were mapped by IPA based on accession numbers. The 270 protein dataset, herein called the “2-fold enriched hBM-MSC-sEV dataset”, is the one presented in this study.

**Additional References:**

1. Benjamini, Y. and Hochberg, Y. (1995). Controlling the false discovery rate: a practical and powerful approach to multiple testing. Journal of the Royal Statistical Society, Series B 57, 289–300.
